# Essential elements of a care delivery model for children with neurological impairments during the COVID-19 pandemic: Notes from Bulgaria

**DOI:** 10.3389/fpubh.2022.932847

**Published:** 2022-08-12

**Authors:** Silviya Pavlova Nikolova, Ruzha Zlatanova Pancheva-Dimitrova, Nikoleta Yoncheva, Virginia Vasileva, Borislava Cherkezova

**Affiliations:** ^1^Department of Social Medicine and Health Care Organisation, Medical University of Varna, Varna, Bulgaria; ^2^Department of Hygiene and Epidemiology, Medical University of Varna, Varna, Bulgaria; ^3^Karin Dom Foundation, Varna, Bulgaria

**Keywords:** coronavirus, Bulgaria, children with neurological impairments, care delivery model, parent-mediated

## Introduction

Children with neurological impairments [NI] and their parents are dealing with extreme challenges resulting from the COVID-19 pandemic. Studies report high mental distress related to restrictions, self-isolation, and quarantines (1–3). In particular, schools and therapeutic centers' closures have placed an excessive burden on families with children with disabilities as home programs for schooling and rehabilitation have not always been accessible in different geographic settings (4, 5). This has forced parents of children with disabilities to juggle multiple roles as teachers, therapists, and caregivers (6). Notably, in the health crisis caused by the COVID-19 pandemic, it is crucial to adopt a model of care, strengthening the role of the family.

A recent review by Novak et al. (7) reports that home-based programs effectively improve the motor function and muscle strength of children with cerebral palsy (7). Additionally, insights from an ongoing Italian family-centered tele-medicine program during the COVID-19 lockdown suggests that personal computers, smartphones and tablets can be successfully used as appropriate tools to respond to the needs of parents and children with special healthcare needs (8). Despite the between-country differences in the management of the coronavirus crisis exist (5, 9, 10), evidence show that appropriately designed home-based programs and innovative technologies can be effectively used to respond to the healthcare needs of children with disability and their families.

This commentary emphasizes the fundamentals of a family-mediated virtual model applied by Bulgarian health and education professionals working with families with children with a disability on how effectively to deliver services to children with neurological impairments during coronavirus disease outbreaks. The model was implemented in Karin Dom, a public outpatient therapeutic center for early intervention services in Varna, Bulgaria. Karin Dom center follows a family-oriented approach that promotes a well-structured environment for group and individual sessions and enhances child's self-control capacities and trust. The family-mediated model of work at Karin Dom was initiated in 1992. It was not until 2020 when the model adopted a remote service delivery of coaching and care. On a usual base, children aged 3-7 years visit the center once or twice a week. The 0–3 age group participate in an early intervention program involving home-based counseling and case management support. The early intervention program includes home visits once or twice a month unless the child needs intensive support or they are part of a crisis intervention program. Depending on the needs of the child and the demands of the parents, the duration of a daily therapy session can vary between 30 min to half a day (4 h, including both group and individual sessions). The usual practice of family-centered approach in the center includes the involvement of parents in the discussion of the individual plan of support of their child, the choice of therapeutic methods, approaches, and strategies of implementation, and is based on the strengths of the family rather than on its deficiencies. As per the remote/online therapy model of work, reliance was heavily focused on role-plays and training of parents on how to work as therapists to their own children. All speech therapists, psychologists, physical therapists, and Montessori educators supported parents by monitoring the implementation of the applied therapeutic methods. The remote partnership between professionals and parents of children with disability focused on strengthening parental capacity and sense of self-efficacy in addressing their child's behavior and unique needs. Additionally, professionals incorporated strategies of building resilience in parents experiencing mental distress and anxiety caused by the home-stay mandates during the pandemic.

## COVID-19 in Bulgaria

The COVID-19 pandemic firstly hit Bulgaria in March 2020. In the beginning, single families, districts, and small localities were only affected. Nine months later, the disease diffused throughout the country and caused an immense increase in the mortality and morbidity rates (11). During the lockdown, parents of children with neurological conditions may have felt alone and neglected in caring for their kids (12) as the country did not adopt a plan to address the needs for specific supportive services of the most fragile individuals, including children with neurological impairments. Therefore, after the suspension of daily rehabilitation services for children with special needs both public and private therapeutic centers started looking for solutions to deliver services.

## Care delivery model for children with neurological impairments during COVID-19 outbreaks

Family-mediated virtual models could be a promising alternative for delivery of services to children with disabilities in times of social and health crises. As a family-centered service model, all specialists involved in family-mediated services, regardless of their professional profile, follows three critical principles of caregiving: (1) information exchange; (2) respectful and supportive care, and (3) partnership (13). Such a family-centered framework is strengths-based and enablement-oriented as it provides important guidelines for health professionals on how best to approach the delivery of services for their clients. At the same time, the framework is flexible and allows to adjust to the behavioral outcomes of the clients as they provide important insights into the practice of health professionals (13, 14).

The family-mediated virtual model [FMVM] used different telecommunication devices such as personal computers, smartphones, and tablets to provide outpatient services and continuity of care. The technological tools' flexible nature allowed practitioners to make assessments and intervention work focused on children with disability and their parents. The model for providing effective remote services to children with NI includes: (1) engagement of parents as mediators of services by establishing a collaborative parent-practitioner partnerships; (2) involving families (parents and children) in decision-making about what and how they would like to improve; (3) active learning strategy and individual training of parents on how to perform different activities to match child's preferences and needs, and unique family routine; (4) providing psychological support to parents aiming to decrease their stress and anxiety; (5) providing psychological support to therapists through weekly supervisions serving different scopes: from development of tailored solutions and strategies of work in online environment to discission of challenges associated with the novel remote treatment delivery in response to the COVID-19 pandemic; (6) assessment of the outcomes by the treatment team.

Research has shown that family-centered and parent-mediated models are equally valued by parents and service providers (14, 15), yet the effectiveness of FMVM is to be determined, as evaluations of evidence-based interventions in the online environment during COVID-19 outbreaks are scarce. A recent survey conducted in the summer of 2020 by the Varna therapeutic center^1^ among 82 families with children with NI revealed that about 10% of the families were reluctant to use social media and remote rehabilitation services. Among the most plausible reasons might have been the negative attitude toward digital technologies and the general belief that face-to-face services are better (16). Additionally, families from low socioeconomic backgrounds, having a limited formal education, living in remote areas with limited internet access could also be part of the underserved families (17). Future programs incorporating FMVM need to promote equality and reduce social disparities in accessing direct services by working closely with local governments and authorities ensuring adequate economic, social, and technical support to children with NI and their families. This is essentially important for countries from the ex-soviet block such as Bulgaria, where national disability policies are not fully standardized nor effectively serving the inclusion of children with disability in the society (11).

The family-mediated virtual model at Karin Dom served to 35 families with children with NI during the first two waves of the SARS-2 coronavirus pandemic in Bulgaria. The center works with a total of 135 families, 100 of which are enrolled in others-not family-mediated therapeutic programs at Karin dom. The FMVM at Karin Dom served with the same intensity and frequency of work during the COVID-19 outbreaks in 2020 as compared to the non-pandemic period of service delivery. Importantly, none of the families refused to work remotely and to cooperate with the professionals at the center. Nevertheless, online rehabilitation to families who were not involved in the family-mediated program before the coronavirus start was challenging, as parents were skeptical that they themselves could effectively collaborate with professionals. Therefore, FMVM for newly engaged families in the program aimed firstly to build a trusting relationship between parents and therapeutic professionals, and secondly to empower parents as active participants in the support model of therapeutic work with their children.

No evidence could support the evaluation of the effectiveness of FMVM at Karin Dom, yet the online survey feedback of 82 parents of children with NI on the impact of the coronavirus service disruption on the overall child rehabilitation strategy and family support collected in May-June, 2020 suggests that 92% of parents were satisfied with the remote way of service delivery. Moreover, more that 80% considered that online rehabilitation support services and counseling did not negatively affect the therapeutic work with their children. Lastly, about 97% of the parents of children with NI found it appropriate to use a hybrid (i.e., online and face-to-face) way of communication and service work with the therapeutic center after the pandemic is over.

## Discussion

Family-mediated models [FMM] of care for children with NI have evolved into variety of forms to meet the needs of the families they treat. Designed to be delivered by people closest to the child, promising FMM have engaged peers (18), siblings (19), and parents (20) and thus allowed to the child to work with people that are likely to impact positively their problem behaviors and skills. Additionally, FMM using technology-mediated communication tools are less likely to face barriers in accessing therapeutic services and are cost-effective (21, 22). Despite being widely used in different countries and settings, evidence on the difference in the effectiveness of FMM of care for children with NI delivered *in situ* or remotely is yet scarce (23). In a systematic review with 14 telehealth interventions involving parents of children with autism, Pacia et al. report that treatment effects for telehealth and *in-situ* interventions were similar, suggesting telehealth as a promising intervention in making gains regardless of whether parents were trained remotely or face-to-face (23). A major limitation of the family-mediated models of care is the underutilization of strategies modifying maladaptive consequences which would rather require a professional involvement on the part of the clinician (23). Additionally, the delivery of remote services of FMM of care may require additional technical training and assistive technology provision for low-income and digitally challenged families (24).

The long road back to normality or the possibility of establishing a more inclusive normality is a future focus. COVID-19 has served as a stark reminder of the persistent social disparities associated with disability. The risk in response to the coronavirus crisis is that children with a disability might be left behind, as health officials' priorities are rather serving general and priority groups and not minorities. To advance social justice and meaningfully include children with disabilities and their families, we need to be bold and rely on principles and strategies that work, such as effective inclusion and equality of opportunities through effective service delivery models. Therefore, innovative approaches such as FMVM that involve family members in the care of children with a disability during the COVID-19 pandemic may be very critical during a crisis as they are expected to lead to improved outcomes and long-lasting progress. They might be the step toward more inclusive normality for children with NI and their families as they make healthcare and education services accessible to underserved communities (25).

## Author contributions

SN and RP-D drafted the manuscript. SN, RP-D, NY, VV, and BC have been involved in the model description. SN and NY in the acquisition of data. All authors contributed to the interpretation of the data, reviewed, and agreed to the final version of this article.

## Conflict of interest

The authors declare that the research was conducted in the absence of any commercial or financial relationships that could be construed as a potential conflict of interest.

## Publisher's note

All claims expressed in this article are solely those of the authors and do not necessarily represent those of their affiliated organizations, or those of the publisher, the editors and the reviewers. Any product that may be evaluated in this article, or claim that may be made by its manufacturer, is not guaranteed or endorsed by the publisher.

## Footnotes

^1^Karin Dom Foundation service center.
